# Monitoring cartilage loss in the hands and wrists in rheumatoid arthritis with magnetic resonance imaging in a multi-center clinical trial: IMPRESS (NCT00425932)

**DOI:** 10.1186/ar4202

**Published:** 2013-03-20

**Authors:** Charles G Peterfy, Ewa Olech, Julie C DiCarlo, Joan T Merrill, Peter J Countryman, Norman B Gaylis

**Affiliations:** 1 Spire Sciences, Inc., 5314 Boca Marina Cir N, Boca Raton, FL, USA; 2 Division of Rheumatology, School of Medicine, University of Nevada, 1707 W. Charleston Blvd, Las Vegas, Nevada, 89102, USA; 3 Oklahoma Medical Research Foundation, 825 NE 13th St, Oklahoma City, Oklahoma, 73104, USA; 4 Arthritis & Rheumatic Disease Specialties, 21097 NE 27th Court, Suite 200, Aventura, Florida, 33180, USA

## Abstract

**Introduction:**

Magnetic resonance imaging (MRI) is increasingly being used in clinical trials of rheumatoid arthritis (RA) because of its superiority over x-ray radiography (XR) in detecting and monitoring change in bone erosion, osteitis and synovitis. However, in contrast to XR, the MRI scoring method that was used in most clinical trials did not include cartilage loss. This limitation has been an obstacle to accepting MRI as a potential alternative to XR in clinical trials. Cross-sectional studies have shown MRI to be sensitive for cartilage loss in the hands and wrist; although, longitudinal sensitivity to change has not yet been confirmed. In this study we examined the ability of MRI to monitor change in cartilage loss in patients with RA in a multi-site clinical trial setting.

**Methods:**

Thirty-one active RA patients from a clinical trial (IMPRESS) who were randomized equally into treatment with either rituximab + methotrexate or placebo + methotrexate had MRI of the dominant hand/wrist at baseline, 12 weeks and 24 weeks at 3 clinical sites in the US. Twenty-seven of these patients also had XR of both hands/wrists and both feet at baseline and 24 weeks. One radiologist scored all XR images using the van der Heijde-modified Sharp method blinded to visit order. The same radiologist scored MR images for cartilage loss using a previously validated 9-point scale, and bone erosion using the Outcome Measures in Rheumatology Clinical Trials (OMERACT) RA MRI Score (RAMRIS) blinded to visit order and XR scores. Data from the two treatment arms were pooled for this analysis.

**Results:**

Mean MRI cartilage score increased at 12 and 24 weeks, and reached statistical significance at 24 weeks. XR total Sharp score, XR erosion score and XR joint-space narrowing (JSN) score all increased at 24 weeks, but only XR total Sharp score increased significantly.

**Conclusions:**

To our knowledge, this is the first publication of a study demonstrating MRI's ability to monitor cartilage loss in a multi-site clinical trial. Combined with MRI's established performance in monitoring bone erosions in RA, these findings suggest that MRI may offer a superior alternative to XR in multi-site clinical trials of RA.

## Introduction

MRI is increasingly being used in clinical trials of rheumatoid arthritis (RA) because of its superiority over conventional radiography (XR) in detecting and monitoring change in bone erosion, osteitis and synovitis [1-5]. Moreover, recent trends in RA research have made XR increasingly impractical as a tool for monitoring disease progression and treatment response. These trends include 1) a shift from conventional placebo-controlled study designs to add-on and active-comparator studies, which require more patients and longer follow-up intervals to discriminate change; 2) the ethical imperative to provide early rescue-therapy to patients showing poor clinical response, which makes detecting structural change quickly a critical need; and 3) a decreasing supply of RA patients suitable for and willing to participate in randomized, controlled studies [6]. As a consequence, no active-comparator trials using XR have been published thus far. Greater sensitivity of MRI to detect change in structural damage, and the ability to demonstrate osteitis and synovitis, provides probative information in less time and with fewer patients than is possible with XR. This has been demonstrated in several recently reported randomized, controlled clinical trials using MRI [1-5]. However, the MRI scoring method used in these studies, the Outcome Measures in Rheumatology (OMERACT) RA MRI score (RAMRIS) [7], did not include assessment of cartilage loss or joint-space narrowing (JSN). This limitation has been an obstacle to substituting MRI for XR in clinical trials, as articular cartilage loss is at least as important, if not more, as bone erosion in determining long-term disability in RA [8], and suppressing bone erosion does not always ensure that cartilage loss has been suppressed as well [2].

Cross-sectional studies have shown MRI to be sensitive for cartilage loss in the hand and wrist, and to correlate well with JSN on XR [9]. However, the longitudinal sensitivity of MRI to detect change in cartilage loss has not yet been confirmed. In this study we monitored change in cartilage loss using conventional 1.5 Tesla (T) MRI in a multi-site clinical trial of patients with active RA.

## Materials and methods

The first 31 patients with active RA (disease duration <5 years) enrolled in a multi-center clinical trial, Impact of Rituximab on Magnetic Resonance Imaging Evidence of Synovitis and Bone Lesions in Patients With Moderate or Severe Rheumatoid Arthritis (IMPRESS), who were randomized equally to treatment with either rituximab + methotrexate or placebo + methotrexate and had MRI of one hand and wrist at baseline, 12 weeks and 24 weeks using the standardized imaging protocol as described below, were included in this analysis. Twenty-seven of these patients also had standardized XR of both hands/wrists and both feet at baseline and 24 weeks. Four of the original 31 patients were missing either baseline or 24-week XR, and therefore were excluded from the XR analysis. The study protocol underwent institutional board review and received ethical approval by The Oklahoma Medical Research Foundation Internal Review Board and the Western Institutional Review Board, and all patients provided informed consent to participate in the study.

### MRI

**Figure 1:**
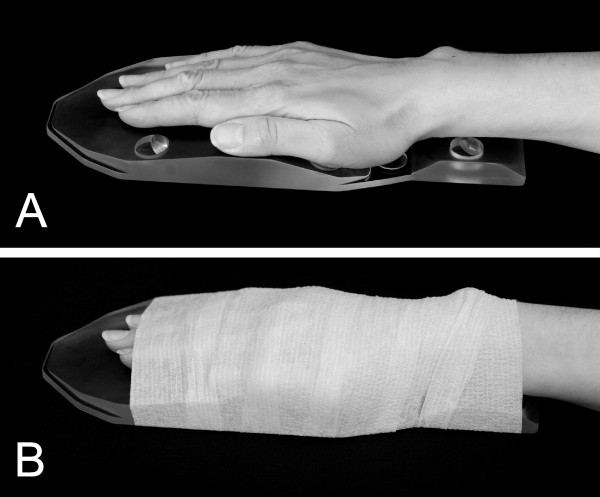
**Positioning device used for reproducible alignment of the bones and joints of the hand and wrist during serial magnetic resonance imaging**. (**A**) Hand and wrist are shown positioned on the acrylic M-frame™ with the fingers and thumb adducted and in plane with each other. (**B**) The hand and wrist are secured to the frame with self-adhesive, latex-free, elastic bandage (images courtesy of Spire Sciences, Inc).

**Figure 2:**
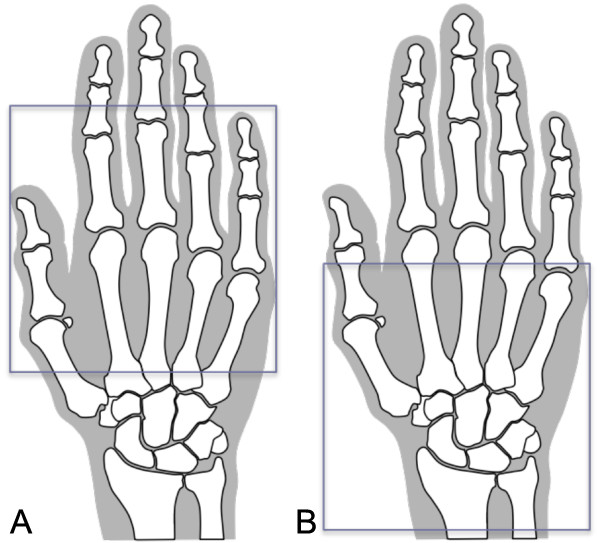
**Positioning of surface coil for magnetic resonance imaging of metacarpophalangeal and proximal interphalangeal joints (A) and wrist joints (B)**. Because of limited field of view of surface coils available at the sites acquiring images for this study, two separate scans (**A**, **B**) were required to cover the hand and wrist completely.

The dominant hand/wrist of each patient was scanned at baseline, 12 weeks and 24 weeks following initiation of therapy, at three clinical sites in the US, using 1.5 T, whole-body MRI and commercial surface coils. Reproducible positioning of the hand/wrist was ensured with a specially designed hand frame (Figure 1). All sites used the same image-acquisition protocol. Only one of the pulse sequences was included in this analysis: coronal, longitudinal magnetic spin relaxation time (T1)-weighted, three-dimensional gradient-echo with spectral fat suppression, but without gadolinium-containing contrast. Repetition time (TR) was 43 ms, echo time (TE) was12 ms, field of view was 120 mm, matrix was 512 × 195 and slice thickness was 1.5 mm, giving a voxel resolution of 234 mm × 625 mm × 1,500 mm. Only one excitation was averaged. Anatomical coverage extended from the distal radioulnar joint proximally to the proximal interphalangeal (PIP) joints distally, and included the entire thumb. Because of the limited field of view of the surface coils used, the joints of the hands were scanned separately from the joints of the wrist (Figure 2). All sites were trained in the image-acquisition protocol prior to study initiation, and all images were centrally evaluated for protocol compliance and image quality by an expert MRI technologist, and were re-acquired if necessary prior to image scoring.

**Figure 3:**
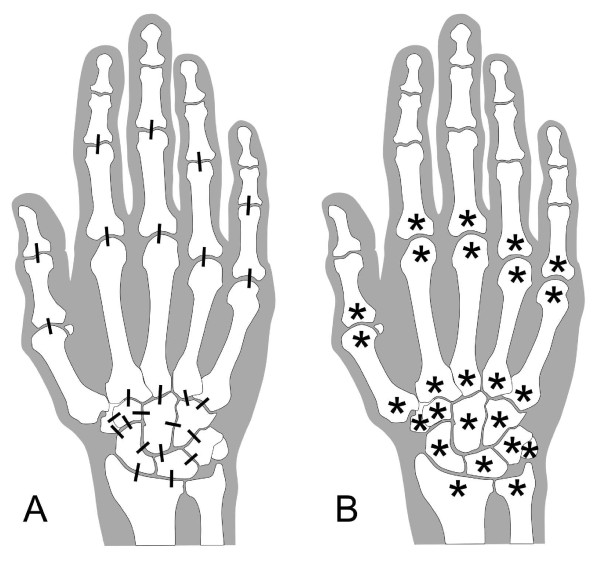
**Locations evaluated for cartilage loss and bone erosion with magnetic resonance imaging**. (**A**) Cartilage loss was scored in proximal interphalangeal joints 1 to 5, metacarpophalangeal joints (MCP) 1 to 5, and 15 joints in the wrist. (**B**) Bone erosion was scored in the 10 bones of MCP 1-5 and all 15 bones of the wrist.

One radiologist (CP) scored all images, blinded to visit order and treatment allocation, using the RAMRIS scale for bone erosion [7] and the previously validated 9-point scale for cartilage loss (MRI cartilage score) [10], where 0.0 = no cartilage loss; 0.5 = equivocal cartilage loss; 1.0 = minimal (<10%) but definitive cartilage loss; 1.5 = mild (10% to 25%) cartilage loss; 2.0 = moderate cartilage loss (26% to 75%, including unilaterally denuded areas but no bilaterally denuded areas or bone-on-bone contact); 2.5 = moderate-severe cartilage loss (>75%, including focal denuding or focal bone-on-bone contact); 3.0 = complete cartilage denuding or diffuse bone-on-bone contact; 3.5 = partial ankylosis; 4.0 = complete ankylosis: the scores are assessed in 25 joints (interphalangeal (IP) 1, PIP 2, PIP 3, PIP 4, PIP 5, metacarpophalangeal (MCP) 1, MCP 2, MCP 3, MCP 4, MCP 5, carpometacarpal (CMC) 2, CMC 3, CMC 4, CMC 5, hamate-capitate, hamate-triquetrum, triquetrum-lunate, capitate-lunate, capitate-scaphoid, capitate-trapezoid, trapezoid-trapezium, scaphoid-trapezium, scaphoid-trapezoid, radius-scaphoid, radius-lunate) (Figure 1b) in each hand/wrist (Figure 3). CMC 1 was excluded because of the high frequency of osteoarthritic cartilage loss in this location. The scapholunate joint was excluded because rupture of the scapholunate ligament often widens this joint. The triquetropisiform joint was excluded because it is not well-visualized in the coronal plane. The distal radioulnar joint was excluded because it is not load bearing and because of difficulty reproducibly aligning the joint on serial MRI. Additionally, this joint was found in previous radiographic studies to be among the least frequently involved locations in the hand and wrist [11].

MRI erosion score and MRI cartilage score from each location in each study patient were summed to determine the MRI total erosion score and MRI total cartilage score at each time point. The maximum possible MRI total erosion score was 250. The maximum possible MRI total cartilage score was 100. MRI total erosion score and MRI total cartilage score were combined to derive the MRI total damage score for each patient at each time point. MRI total damage score was corrected for differences in the scales for MRI erosion score and MRI cartilage score, such that MRI total damage score = MRI total erosion score + (2.5 × MRI total cartilage score). The maximum possible MRI total damage score was thus 500. All analyses were based on these patient-level total scores.

### Radiography

Each of the two hands/wrists and feet of the 27 patients included in the XR analysis were radiographed separately on high-resolution 10 × 12 inch, single-emulsion, single screen film, using standardized positioning with a template. Hands/wrists were exposed posterior-anteriorly with the beam centered between the second and third MCP joints and perpendicular to the cassette. Radiographs were digitized to a pixel size of 100 mm at 12 bits per pixel. One radiologist (CP) scored all images, blinded to visit order and MRI results, for erosion and JSN using the van der Heijde-modified Sharp method [12].

Statistical significance of change in XR or MRI score from baseline to 12 weeks or 24 weeks was assessed using the two-sided *t*-test, with *P *= 0.05 as the cutoff for significance. Data from the two treatment arms were pooled for this analysis in order to maintain blinding to treatment, as the study was still ongoing.

## Results

Baseline characteristics of the patients were as follows: mean disease duration was <5 years; mean age was 42.2 years; 24 patients were women; 7 patients were men; mean disease duration was 21 months; 84% of patients were rheumatoid factor (RF)-positive; 65% were anti- cyclic citrullinated peptide (CCP)-positive; mean disease activity score (DAS)28 erythrocyte sedimentation rate (ESR) was 6.74.

**Figure 4:**
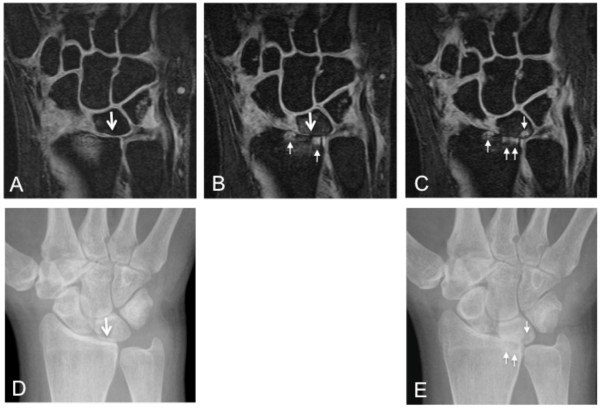
**Progression of joint damage in the radiolunate joint**. Magnetic resonance imaging (MRI) of the wrist shows thin but present high-signal-intensity cartilage over articular surfaces of the radiolunate joint (large arrow) at baseline (**A**) associated with grade-1.0 cartilage thinning. Corresponding baseline conventional radiography (XR) (**D**) shows joint-space narrowing (JSN) at this location (large arrow). Follow-up MRI at 12 weeks (**B**) and 24 weeks (**C**) show progressive JSN associated with loss of cartilage over both articular surfaces of this joint, indicative of grade-3.0 cartilage loss. Note development and progression of bone erosions (small arrows) in the radius and lunate on these follow-up scans. Week-24 XR also shows complete radiolunate JSN, the lunate erosion and two of the radius erosions (small arrows). However, the third erosion in the radius is not visible on XR.

**Figure 5:**
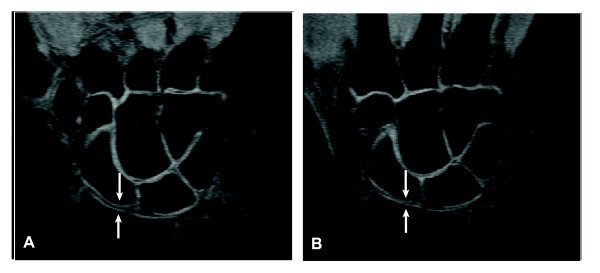
**Assessing cartilage directly is more accurate than measuring joint-space width**. Intact articular cartilage can be seen in these magnetic resonance images of the wrist as bands of high-signal-intensity tissue lining the surfaces of the bones and showing sharp contrast with low-signal-intensity joint fluid along the articular surfaces, and very low-signal-intensity bone cortex and suppressed marrow fat along the subchondral surfaces. Follow-up image (**B**) shows narrowing of the radioscaphoid joint space (arrows) relative to that in image (**A**). However, this narrowing is the result of displacement of joint fluid from between the intact articular cartilage surfaces because of abduction of the wrist rather than because of thinning of the cartilage plates themselves. Thus, joint-space width can be an inaccurate measure of cartilage thickness.

**Figure 6:**
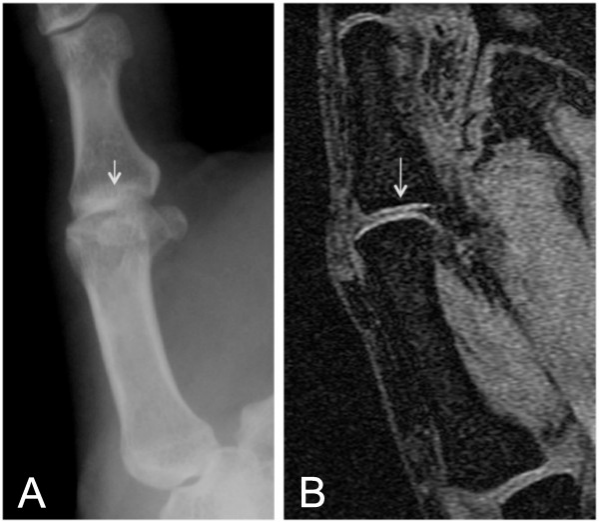
**Oblique radiographic projection can simulate joint-space narrowing on conventional radiography (XR)**. (**A**) Metacarpophalangeal joint (MCP)-1 joint space (short arrow) appears narrowed due to oblique projection on XR. (**B**) Magnetic resonance imaging of the same MCP joint shows intact articular cartilage and normal joint-space width (long arrow).

**Figure 7:**
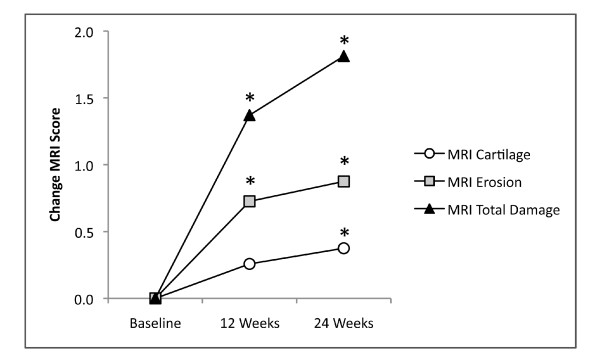
**Mean change in magnetic resonance imaging (MRI) scores (one hand per patient) from baseline**. *P <0.05.

**Figure 8:**
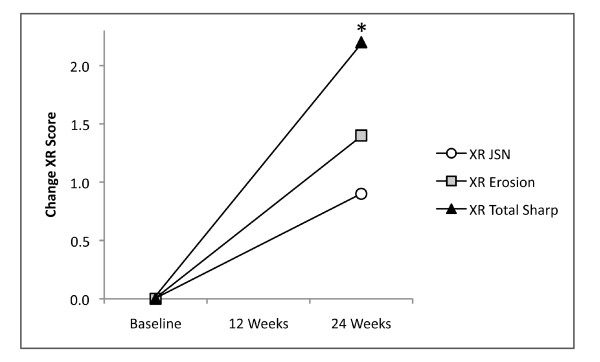
**Mean change in conventional radiography (XR) scores (two hands, wrists, feet per patient) from baseline**. *P <0.05.

Of 2,325 joints assessed with MRI, 4% were outside the field of view and could not be assessed. The majority of these were PIP joints. Bone erosion and cartilage loss were both well-seen on fat-suppressed coronal, T1-weighted, three-dimensional, gradient-echo images (Figures 4, 5 and 6). As shown in Figure 7, mean total cartilage score increased from 4.2 at baseline to 4.5 at 12 weeks (mean change ± SD = 0.3 ± 0.9, *P *= 0.124), and significantly to 4.6 at 24 weeks (mean change ± SD = 0.4 ± 1.1, *P *= 0.034). MRI total erosion score increased significantly from 7.9 at baseline to 8.7 at 12 weeks (mean change ± SD = 0.7 ± 5.7, *P *= 0.024), and 8.8 at 24 weeks (mean change ± SD = 0.9 ± 5.7, *P *= 0.030). MRI total damage score showed a similar pattern with significant change from 18.5 at baseline to 19.9 at 12 weeks (mean change ± SD = 1.4 ± 19.1, *P *= 0.050), and 20.4 at 24 weeks (mean change ± SD = 1.98 ± 19.8, *P *= 0.011). The same hand showed similar but non-significant XR progression from baseline to 24 weeks (XR erosion score change ± SD = 0.6 ± 1.8, *P *= 0.174; JSN score change ± SD = 0.4 ± 1.3, *P *= 0.181; total sharp score change ± SD = 1.0 ± 3.1, *P *= 0.181). Radiography of both hands/wrists and feet also showed non-significant progression in the erosion score (mean change ± SD = 1.4 ± 3.7, *P *= 0.58) and JSN score (mean change ± SD = 0.9 ± 2.9, *P *= 0.104), but significant change in the total sharp score of bilateral hands, wrists and feet (mean change ± SD = 2.2 ± 6.3, *P *= 0.034) at 24 weeks (Figure 8).

## Discussion

Cross-sectional studies [9] have shown MRI to be sensitive to cartilage loss and JSN in RA, but to our knowledge this is the first publication of a study demonstrating the ability of MRI to monitor longitudinal change in cartilage loss in a time-blinded, multi-site clinical trial.

Significant progression of cartilage loss was demonstrated in this study within 24 weeks with only a single hand/wrist of 31 patients. Moreover, combining total cartilage score with total erosion score allowed determination of a MRI total damage score (Figure 7), analogous to that used in XR Sharp scoring [13]. Progression of bone erosion and JSN in these patients was confirmed on XR, but statistical significance with XR was reached only for total sharp score by 24 weeks and only when bilateral hands, wrists and feet were included in the analysis.

Including cartilage loss in MRI assessment of RA is important because this feature of joint destruction does not always follow the same pattern of response to therapy as bone erosion does. Indeed, separating JSN and bone erosion was a fundamental innovation introduced in XR Sharp scoring [11], over global scoring methods, such as the Larson method [14]. This disconnect between cartilage loss and bone erosion is illustrated in the randomized, controlled trial of denosumab, reported by Cohen *et al*. [2], in which, 227 patients with established RA were treated with either placebo plus methotrexate, or one of two doses of denosumab plus methotrexate, and followed longitudinally with XR (van der Heijde-Sharp erosion and JSN scoring) and MRI (RAMRIS erosion scoring). Whereas both RAMRIS and XR Sharp scoring showed denosumab to have strong erosion-suppressing effects, XR showed denosumab to have no effect on preventing JSN. Had only RAMRIS been used, the lack of efficacy on cartilage loss would not have been detected.

Accordingly, the OMERACT MRI Working Group, which originally developed RAMRIS, recently proposed adding a JSN component [15]. The proposed MRI-JSN score uses a five-point scale similar but not identical to the van der Heijde Sharp XR JSN scale, and was shown to correlate with XR scoring cross-sectionally. McQueen *et al*. [11] also compared MRI to XR using a five-point scale to assess the wrists of 38 patients with RA and 22 control subjects. Although the joints evaluated by MRI and XR in their study were not exactly the same as those in either the van der Heijde-modified or the Genant-modified Sharp XR methods, correlations between the MRI cartilage score in the wrist and total XR JSN score were high in the same wrist (0.61 to 0.74) and in both hands, wrists and feet (0.68 to 0.78).

The nine-point MRI cartilage score [10] used in this study was modeled after the Genant-modified Sharp XR JSN score [13], which has been used in multiple clinical trials to gain regulatory approval of structure-modifying therapies, including abatacept [12], rituximab [16] and tocilizumab [17]. Since the nine-point MRI scale contains more increments than five-point scales, it may have an advantage in terms of sensitivity to change; however, this has not been tested directly. Direct comparisons of the five-point van der Heijde-modified Sharp and nine-point Genant-modified Sharp XR JSN scoring methods, however, found both methods to have the same discriminative power for XR detection of change in JSN over time, and between treatment arms [18,19]. Because only a single reading of the images was performed in this study, the intra-reader and inter-reader variability of the nine-point MRI cartilage score is not known.

That MRI is good at monitoring cartilage loss in RA is not surprising. First, the MRI tomographic viewing perspective obviates projection of the rims of the concave articular surfaces of joints over the joint space, which can mimic JSN on conventional radiographs (Figure 6) [20]. Additionally, ligamentous laxity/rupture and interposition of synovial tissue or joint effusion between articular surfaces can decrease the accuracy of XR JSN as a measure of cartilage loss (Figure 5). Thus, the ability of MRI to visualize articular cartilage directly rather than only on the basis of the width of the space between opposing articular cortices is a substantial advantage [21-25]. The MRI protocol used in this study is the same as that used for monitoring bone erosion with RAMRIS in many other clinical trials of RA [1-5]. Thus, MRI protocols do not need to be expanded in order to add MRI cartilage score to assessments of joint damage.

Fat-suppressed, T1-weighted, three-dimensional, gradient-echo scans have been shown to delineate articular cartilage accurately in various joints, including the MCPs [22], and are commercially available on all clinical MRI systems operating at magnetic field strengths of 1.0 T or higher. Systems operating at lower field strengths currently have difficulty with this technique because of limitations in spectral fat suppression or selective water excitation. Selective fat suppression or water excitation is important for increasing T1 contrast between cartilage and adjacent joint fluid or subchondral bone (marrow fat) and for eliminating chemical-shift effects [23], which distort cartilage-bone interfaces and can simulate cartilage thinning and JSN. Increasing receiver bandwidth can reduce chemical shift, but this reduces the signal-to-noise ratio of the images, and does not completely eliminate the problem.

## Conclusions

In conclusion, the findings presented in this study, taken in combination with those from prior studies validating cartilage assessment with MRI against JSN scoring with XR, suggest that MRI may offer a superior alternative to XR in multi-site clinical trials of RA. With the recent shift towards active-comparator study designs, which require longer study durations and more patients to demonstrate therapeutic superiority, and the increasing difficulty in recruiting RA patients into clinical trials, there is a growing need for more sensitive methods, such as MRI, to offset the escalating costs, patient exposure and logistical challenges associated with these trends.

## Abbreviations

CCP: cyclic citrullinated peptide; CMC: carpometacarpal; DAS: disease activity score; ESR: erythrocyte sedimentation rate; IP: interphalangeal; JSN: joint-space narrowing; MCP: metacarpophalangeal; MRI: magnetic resonance imaging; OMERACT: Outcome Measures in Rheumatology; PIP: proximal interphalangeal; RA: rheumatoid arthritis; RAMRIS: rheumatoid arthritis magnetic resonance imaging score; RF: rheumatoid factor; T: Tesla; T1: longitudinal magnetic spin relaxation time; TE: echo time; TR: repetition time; XR: x-ray.

## Competing interests

Charles G Peterfy: shareholder of Spire Sciences Inc., past shareholder of Synarc Inc., Consultant for AbbVie, Amgen, AstraZeneca, Bristol Myers-Squibb, BioClinica, Celgene, Centocor, Eli Lilly and Company, Genentech, Genzyme, Icon Medical Imaging, Johnson & Johnson, Medimmune, Merck, Novartis, Perceptive Informatics, Pfizer, Rigel, Roche, Sanofi, Samsung, Santarus, UCB, VirtualScopics, Wyeth, employee of Spire Sciences Inc., past employee of Synarc Inc, Speakers Bureau of Amgen. Ewa Olech: grant/research support from Genentech, Consultant for Genentech, Speakers Bureau of Genentech. Julie Camille DiCarlo: Consultant for Abbvie, Amgen, AstraZeneca, Bristol Myers-Squibb, BioClinica, Celgene, Centocor, Core Lab Partners, Crescendo, Eli Lilly and Company, Genentech, Genzyme, Medimmune, Merck, Icon Medical Imaging, Novartis, Perceptive Informatics, Pfizer, Rigel, Roche, Sanofi, Santarus, UCB, VirtualScopics, Wyeth, employee of Spire Sciences Inc., and past employee of Synarc Inc. Joan T. Merrill: grant/research support from Genentech, Consultant for Genentech, Speakers Bureau of Genentech. Peter Countryman: employee of Spire Sciences. Norman Gaylis: grant/research support from Genentech, Consultant for Genentech, Speakers Bureau of Genentech.

## Authors' contributions

CP participated in protocol and experimental design, radiological analysis, and drafted the manuscript. EO participated in protocol design, imaging acquisition, and reading results analysis. JD participated in protocol and experimental design, imaging acquisition and analysis of the reading results, and helped draft the manuscript. JTM participated in protocol design, imaging acquisition, and reading results analysis. PC participated in protocol design, imaging acquisition, and reading results analysis. NG participated in protocol design, imaging acquisition, and reading results analysis. All authors read and approved the final manuscript.
